# Phytocannabinoids and Nanotechnology in Lung Cancer: A Review of Therapeutic Strategies with a Focus on Halloysite Nanotubes

**DOI:** 10.3390/ph18091244

**Published:** 2025-08-22

**Authors:** Dorota Bęben, Helena Moreira, Ewa Barg

**Affiliations:** Department of Basic Medical Sciences, Faculty of Pharmacy, Wroclaw Medical University, 50-556 Wroclaw, Poland

**Keywords:** lung cancer, nanocarriers, halloysite nanotubes, phytocannabinoids, targeted drug delivery

## Abstract

Lung cancer is the leading cause of cancer mortality worldwide, with a poor prognosis driven by late diagnosis, systemic toxicity of existing therapies, and rapid development of multidrug resistance (MDR) to agents such as paclitaxel and cisplatin. MDR arises through multiple mechanisms, including overexpression of efflux transporters, alterations in apoptotic pathways, and tumour microenvironment-mediated resistance. The application of nanotechnology offers a potential solution to the aforementioned challenges by facilitating the enhancement of drug solubility, stability, bioavailability, and tumour-specific delivery. Additionally, it facilitates the co-loading of agents, thereby enabling the attainment of synergistic effects. Halloysite nanotubes (HNTs) are naturally occurring aluminosilicate nanocarriers with unique dual-surface chemistry, allowing hydrophobic drug encapsulation in the positively charged lumen and functionalisation of the negatively charged outer surface with targeting ligands or MDR modulators. This architecture supports dual-delivery strategies, enabling simultaneous administration of phytocannabinoids and chemotherapeutics or efflux pump inhibitors to enhance intracellular retention and cytotoxicity in resistant tumour cells. HNTs offer additional advantages over conventional nanocarriers, including mechanical and chemical stability and low production cost. Phytocannabinoids such as cannabidiol (CBD) and cannabigerol (CBG) show multitarget anticancer activity in lung cancer models, including apoptosis induction, proliferation inhibition, and oxidative stress modulation. However, poor solubility, instability, and extensive first-pass metabolism have limited their clinical use. Encapsulation in HNTs can overcome these barriers, protect against degradation, and enable controlled, tumour-targeted release. This review examined the therapeutic potential of HNT-based phytocannabinoid delivery systems in the treatment of lung cancer, with an emphasis on improving therapeutic selectivity, which represents a promising direction for more effective and patient-friendly treatments for lung cancer.

## 1. Introduction

Lung cancer remains the leading cause of cancer-related mortality worldwide, accounting for over 1.8 million deaths each year, approximately 18% of all cancer-related deaths [1]. Despite intensive preventive measures, the global incidence of this malignancy remains significant. While a decline in incidence has been observed among men in developed countries, the trend is less pronounced or even increasing among women. This trend indicates that lung cancer may increasingly affect women in the coming decades if current risk factors persist, particularly in the absence of effective preventive measures and consideration of genetic predisposition. However, future changes in exposure to these risk factors could potentially reverse this pattern. Tobacco smoking remains the primary risk factor. However, the increasing number of cases among non-smokers underscores the growing significance of environmental contributors, such as exposure to fine particulate matter (PM_2.5_), occupational hazards, and genetic predisposition [2,3,4].

The therapeutic approach to lung cancer is determined by its histological subtype and stage of progression. There are two major subtypes: non-small-cell lung cancer (NSCLC), which constitutes the majority of cases and allows for broader treatment options, and small-cell lung cancer (SCLC), which exhibits aggressive clinical behaviour and a poorer prognosis. NSCLC can be treated with surgery, chemotherapy, radiotherapy, targeted therapies, and immunotherapy. In contrast, SCLC is predominantly treated with a combination of chemotherapy and radiotherapy, which often has limited efficacy and results in a poor prognosis.

Significant advances have been made in recent years through the implementation of molecularly targeted therapies such as epidermal growth factor receptor (EGFR), anaplastic lymphoma kinase (ALK), c-ros oncogene 1 (ROS1), and B-Raf proto-oncogene (BRAF) inhibitors and immune checkpoint inhibitors: programmed cell death protein 1 (PD-1)/programmed death-ligand 1 (PD-L1). These advances have significantly improved patient survival and quality of life [5,6,7,8,9].

Nevertheless, the effectiveness of current oncological therapies is increasingly being hindered by the emergence of multidrug resistance (MDR). This phenomenon often involves ATP-binding cassette (ABC) efflux transporters, such as P-glycoprotein (P-gp/ABCB1) and multidrug resistance-associated proteins (MRPs), which actively expel chemotherapeutic agents from cancer cells, thereby reducing their intracellular concentrations and therapeutic efficacy [9,10]. In non-small-cell lung cancer (NSCLC), resistance to agents such as paclitaxel (Taxol^®^) and cisplatin often involves epithelial–mesenchymal transition (EMT), activation of PI3K/AKT/mTOR and MAPK pathways, and alterations in microtubule-regulating proteins. Taxol resistance has been linked to extracellular signal-regulated kinase (ERK)-mediated phosphorylation of oncoprotein 18 (Op18)/stathmin, which disrupts microtubule dynamics and reduces drug sensitivity. Inhibition of ERK restores Taxol sensitivity by promoting apoptosis, reducing Op18/stathmin phosphorylation, and downregulating anti-apoptotic proteins such as Bcl-2 [11].

Recent studies have shown that fluorofenidone (AKF-PD) significantly inhibits NSCLC cell proliferation and invasion, suppresses MAPK and PI3K/AKT/mTOR signalling, and reverses EMT. Importantly, AKF-PD enhances the antitumour efficacy of cisplatin while simultaneously mitigating cisplatin-induced nephrotoxicity. The combination of AKF-PD with cisplatin produced stronger inhibition of tumour cell growth and migration compared to either agent alone, both in NSCLC and in other cancer cell types [12].

These mechanisms contribute to overcoming drug resistance and improving therapeutic outcomes. Furthermore, the systemic toxicity associated with conventional treatments limits the use of higher drug doses and represents a significant source of adverse effects. The development of clinical resistance and the poor selectivity of anticancer drugs highlights the urgent need for novel therapeutic strategies that are more effective and less harmful to healthy tissues [13,14].

Replacing or complementing conventional chemotherapeutic agents with phytomedicine-derived compounds, such as phytocannabinoids, is a promising strategy for overcoming certain resistance mechanisms. Cannabinoids have been shown to modulate multiple oncogenic and survival pathways, induce apoptosis, and promote oxidative stress in cancer cells, This could potentially overcome drug resistance when used as a single treatment [15].

Furthermore, oxidative stress—defined as an imbalance between the production of reactive oxygen species (ROS) and the antioxidant defence system—plays a dual role in lung cancer. Chronic oxidative stress contributes to DNA damage, genomic instability, and tumour progression, while controlled induction of ROS can trigger apoptosis and inhibit tumour growth. This dual nature makes oxidative stress both a pathogenic driver and a therapeutic target, particularly in approaches combining ROS modulation with targeted delivery systems.

One of the most promising avenues for anticancer treatment is nanomedicine, which uses nanocarriers to deliver therapeutic agents to specific sites. This approach increases the accumulation of active compounds within tumour sites while reducing exposure to healthy tissues, thereby minimising adverse effects. Nanocarriers such as liposomes, polymeric nanoparticles, or clay-based nanotubes can be functionalised for active targeting and controlled release, improving pharmacokinetics and helping to overcome multidrug resistance.

The present review aims to provide a comprehensive analysis of the current knowledge on the application of nanotechnology in the treatment of lung cancer, with particular focus on the therapeutic potential of phytocannabinoids and their integration into nanocarrier-based systems. The potential synergies between phytocannabinoids and natural nanocarriers, such as halloysite nanotubes (HNTs), may provide a new therapeutic approach to managing this deadly disease.

## 2. Oxidative Stress in Lung Cancer

Oxidative stress is defined as an imbalance between the production of reactive oxygen species (ROS) and reactive nitrogen species (RNS) and the capacity of antioxidant defence systems to neutralise them. Although low to moderate levels of ROS and RNS are necessary for physiological processes such as cell signalling, gene regulation, and the defence of the host, excessive or sustained ROS production can damage cellular components, including lipids, proteins, and DNA, and it can ultimately contribute to the development of cancer [16,17].

The lungs are particularly vulnerable to oxidative damage due to their constant exposure to endogenous oxidants and exogenous sources such as air pollutants, cigarette smoke, respirable particulate matter (PM_2.5_, PM_10_), fibrous dust, and ozone. These agents can generate reactive oxygen species (ROS) directly or indirectly, thereby initiating inflammatory cascades and activating signalling pathways such as mitogen-activated protein kinases (MAPK), nuclear factor kappa-light-chain-enhancer of activated B-cells (NF-κB), and activator protein-1 (AP-1). These pathways are involved in processes such as cell proliferation, apoptosis resistance, and tumour progression [17].

Oxidative stress is closely linked to chronic pulmonary inflammation, which is a recognised driver of lung cancer development. Inflammatory cells, particularly macrophages and neutrophils, produce large amounts of reactive oxygen species (ROS) and reactive nitrogen species (RNS) via enzymatic systems such as NADPH oxidase and inducible nitric oxide synthase (iNOS). This creates a highly oxidative microenvironment that can induce oxidative DNA lesions such as 8-hydroxy-2′-deoxyguanosine (8-OHdG) and promote genomic instability and malignant transformation [18].

Crucially, cancer cells can adapt to elevated ROS levels by increasing the activity of endogenous antioxidant systems, such as glutathione (GSH), superoxide dismutase (SOD), catalase (CAT), and the thioredoxin (Trx) system. This adaptive shift enables tumour cells to tolerate oxidative stress and exploit ROS for pro-survival signalling and metastasis. Therapeutic strategies that disrupt this redox balance by either increasing ROS beyond the toxic threshold or inhibiting antioxidant defences have shown promise in preclinical models [18,19].

Given its dual role in tumour biology—contributing to both cancer initiation and progression, while also serving as a potential therapeutic target—oxidative stress represents a critical mechanistic link between environmental risk factors and lung cancer pathogenesis. Targeting ROS-mediated pathways offers opportunities for therapeutic intervention, particularly through strategies that modulate redox balance. This includes the use of phytochemicals, such as cannabinoids, which can influence oxidative status by either inducing ROS-dependent cancer cell death or mitigating oxidative damage in non-malignant tissues. This could be particularly effective when phytochemicals are delivered via nanocarrier systems.

Understanding the relationship between oxidative stress and the pathogenesis of lung cancer provides a foundation for designing targeted therapeutic strategies. Nanotechnology-based delivery systems can be engineered to enhance ROS generation selectively within tumour tissues or deliver antioxidant agents to protect healthy lung tissue. In this context, phytocannabinoids are an attractive option as they can modulate redox balance.

## 3. Nanocarriers in Anti-Cancer Therapies

In recent years, a wide array of nanocarrier systems have been developed in the field of translational medicine, particularly in oncology. Among the most widely studied are lipid-based nanocarriers, including liposomes and solid lipid nanoparticles (SLNs), both characterised by a spherical architecture formed by a phospholipid bilayer. A major advantage of these carriers lies in their ability to prolong the presence of therapeutics in systemic circulation through PEGylation, while simultaneously reducing drug-associated toxicity. Notable examples include liposomal doxorubicin (Doxil^®^/Caelyx^®^), approved by the U.S. Food and Drug Administration (FDA) for the treatment of breast cancer and Kaposi’s sarcoma [20,21,22]. Liposomal oxaliplatin (MBP-426) is currently undergoing Phase II clinical trials. The MITO-Porter system has been developed specifically to target the mitochondria of cancer cells [22].

Polymeric micelles are another important class of nanocarriers. These amphiphilic structures consist of a hydrophobic core for encapsulating poorly water-soluble drugs and a hydrophilic polyethylene glycol (PEG) shell for ensuring colloidal stability and prolonged circulation. One example is Genexol-PM (micellar paclitaxel), currently under clinical investigation [21]. Polymeric nanoparticles composed of materials such as polylactic acid (PLA), polylactic-co-glycolic acid (PLGA), and polyglycolic acid (PGA) are equally significant due to their ability to degrade in a controlled manner, thereby enabling sustained drug release. Examples with clinical relevance include CALAA-01 (PLGA-siRNA) and BIND-014, a docetaxel formulation conjugated with a prostate-specific membrane antigen (PSMA)—targeting ligands used in prostate cancer therapy [22]. Protein-based nanoparticles, such as albumin-bound paclitaxel (Abraxane^®^), also have applications in cancer therapy. They demonstrate excellent biocompatibility and have been approved by the FDA for the treatment of various cancers [21,23].

Inorganic nanocarriers, including gold nanoparticles (AuNPs) and superparamagnetic iron oxide nanoparticles (SPIOs, e.g., Ferumoxide), have emerged as key components in theranostic applications. For instance, AuNPs conjugated with transferrin facilitate the transport of drugs across the blood–brain barrier, whereas SPIOs serve both as drug delivery platforms and as contrast agents in MRI imaging [21].

Carbon-based nanostructures, such as single-walled carbon nanotubes (SWCNTs) and quantum dots (QDs), are notable for their high surface area and photothermal properties. Despite their promising diagnostic and therapeutic potential, their clinical translation is hindered by toxicity concerns [23].

In recent developments, sophisticated multistage and organelle-targeting nanocarriers have been engineered to respond to tumour-specific microenvironmental cues, such as pH or reactive oxygen species (ROS). This enables the spatial and temporal control of drug release. Examples include pH-responsive gold–DNA nanoclusters and chondroitin-based nanomicelles designed to target the Golgi apparatus [24].

### Nanocarrier-Based Delivery Systems for Lung Cancer Treatment

The development of novel therapeutic strategies has underscored the importance of nanocarriers in lung cancer treatment. While some systems are actively targeted through ligand functionalisation to enhance tumour specificity, others, such as non-targeted polymeric nanoparticles or liposomes, contribute by enabling controlled drug release, improving pharmacokinetics, and reducing systemic toxicity. Particularly significant are polymeric nanoparticles made from biocompatible materials such as polylactic acid (PLA), poly (lactic-co-glycolic acid) (PLGA), and chitosan. These materials enable controlled drug release and are well-suited for targeted drug delivery. Polymeric nanoparticles are employed in both gene therapy and chemotherapy for non-small-cell lung cancer (NSCLC) through active receptor-mediated targeting, via receptors such as the epidermal growth factor receptor (EGFR), transferrin, or integrins, as well as passive targeting via the enhanced permeability and retention (EPR) effect. This approach has been exemplified by PLGA-siRNA systems used in experimental therapies against NSCLC [25].

Lipid-based nanocarriers, such as liposomes and solid lipid nanoparticles (SLNs), enhance drug bioavailability and reduce the toxicity of chemotherapy through active targeting (e.g., EGFR, transferrin receptors) and passive EPR accumulation. Liposomal doxorubicin and oxaliplatin are currently under investigation for NSCLC [26]. Due to their higher physical stability and more favourable pharmacokinetics, SLNs may outperform conventional liposomes in delivering hydrophobic agents such as paclitaxel [25].

Metal-based nanoparticles, such as those composed of gold or silver, are increasingly utilised for their unique optical and photothermal properties. These nanoparticles are actively targeted via surface ligand conjugation and are primarily investigated for photothermal therapy to complement standard lung cancer treatments [27].

Modern strategies also incorporate hybrid organic–inorganic nanocarriers, which combine the properties of metallic and polymeric systems synergistically. These hybrid platforms allow multimodal therapeutic targeting (e.g., via integrins and EGFR) and simultaneous chemo- and photothermal therapy. This enhances tumour penetration and therapeutic efficacy. Current clinical applications predominantly focus on these combinatorial therapeutic modalities [28].

Another promising approach involves biomimetic nanocarriers, which are nanoparticles coated with membranes derived from cancer or immune cells. This phenomenon refers to the ability of membrane-coated nanoparticles to preferentially bind to cancer cells of the same type as their membrane origin, thereby enhancing selective accumulation in tumour tissue. In addition, these carriers can support immunomodulation, offering a route toward more personalised treatment regimens for lung cancer. However, challenges such as standardisation of production and potential immunogenicity remain key limitations to their applicability in practice [22].

Furthermore, active investigations are underway into tumour microenvironment (TME)-responsive nanocarriers. These systems have been engineered to respond to pathological stimuli present within the tumours, such as reduced pH, hypoxia, or elevated reactive oxygen species (ROS). Enabling condition-specific drug release by TME-targeted nanocarriers has been shown to improve the precision and efficacy of therapy, supporting both immunotherapy and chemotherapy interventions [29]. Table 1 provides an overview of the nanocarriers examined in lung cancer.

## 4. Biomedical Applications of Halloysite Nanotubes in Targeted Drug Delivery

The variety of nanocarrier strategies currently being investigated indicates the broad therapeutic potential of nanomedicine in the field of oncology. Alongside the substantial research conducted on lipid-, polymer-, and metal-based nanoparticles, there has been a growing focus on systems based on natural minerals. Among these, halloysite—a naturally occurring aluminosilicate with a nanotubular structure—has emerged as a promising candidate due to its abundance, high specific surface area, low toxicity, and strong drug-encapsulation capabilities. These properties place HNTs as cost-effective and efficient nanocarriers for anticancer applications.

### 4.1. Characteristics of Halloysite Nanotubes (HNTs)

**Figure 1:**
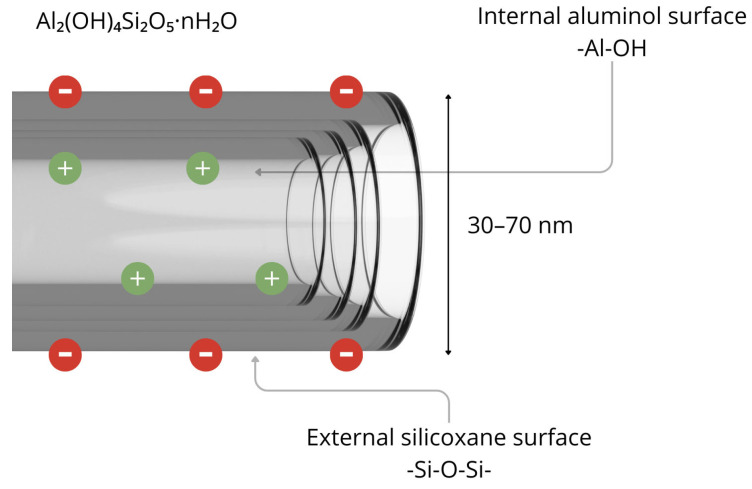
Structure of halloysite nanotube. Figure created by the authors.

Halloysite nanotubes (HNTs) are naturally occurring aluminosilicate minerals with the general chemical formula Al_2_(OH)_4_Si_2_O_5_·nH_2_O. HNTs exhibit a cylindrical morphology, with diameters ranging from 30 to 70 nm and lengths up to 1500 nm. This distinguishes them as one of the few naturally occurring nanomaterials with a tubular structure (Figure 1) [30].

In their native form, HNTs have a high specific surface area ranging from 50 to 70 m^2^/g, which can be further increased by up to 17.5% through surface functionalisation [31]. They also offer substantial pore volume. The inner lumen of HNTs carries a positive charge, favouring the adsorption of anionic compounds, while the outer surface is amenable to a wide range of chemical modifications [32]. From a biomedical perspective, HNTs are widely regarded as biocompatible and exhibit low biological toxicity, making them highly attractive for use in pharmaceuticals and medicine [33]. Their tubular architecture facilitates the encapsulation of active pharmaceutical ingredients within the inner lumen, supporting sustained release over time. For example, the release of cinnamaldehyde essential oil from HNTs has been shown to remain stable for more than seven days [34]. Functionalizing HNTs with metal ions (e.g., Ag, Fe_2_O_3_) enhances their utility by imparting antimicrobial properties and improving adsorption efficiency. Silver-modified HNTs, for example, have notable antibacterial properties and are substantially effective against a range of Gram-positive and Gram-negative bacterial strains [31]. Another major advantage lies in the chemical and mechanical stability of halloysite, which is derived from its multilayered structure. Its resistance to acidic and alkaline conditions, along with robust mechanical strength, enables HNTs to withstand harsh technological and environmental conditions during formulation and use [31]. Furthermore, the outer surface of HNTs can be conjugated with targeting ligands such as folic acid, monoclonal antibodies, or peptides, enabling precise drug delivery in oncology and inflammatory disease settings. These targeted systems enhance pharmacokinetic properties and safety profiles, thus supporting the development of advanced nanotherapeutic platforms [32].

### 4.2. Applications of HNTs as Anticancer Drug Carriers

A promising approach to the use of HNTs as anticancer drug carriers involves hybrid formulations using HNTs in combination with copper oxide and curcumin (CHC). In vitro studies have demonstrated their cytotoxic activity against the melanoma (HMVII) cell line, as well as liver (HepG2) and breast (MCF-7) cancer cell lines. After 24 h, the system showed an IC_50_ of 10.43 μg/mL, indicating the synergistic efficacy of CuO and curcumin in inhibiting proliferation and inducing apoptosis [35].

Equally promising are HNTs functionalised with silver nanoparticles (AgNPs), synthesised via green methods. Green synthesis is an environmentally friendly approach to nanoparticle fabrication that uses plant extracts or other biological agents as natural reducing and stabilising compounds. This green–functionalised system demonstrated strong cytotoxicity against Jurkat T-cell leukaemia cells (IC_50_ of 1.77 μg/mL), through apoptosis induction (upregulation of caspase-3, increased Bcl-2 homologous antagonist/killer 1 / B-cell lymphoma-extra large [Bak1/Bclx] ratio) as well as oxidative stress [36]. A significant number of studies have also focused on doxorubicin (DOX)-based HNT formulations. Functionalisation with ligands such as folic acid (FA), polyethylene glycol (PEG), or aptamers enables precise targeting of breast (MCF-7), cervical (HeLa), or lung (A549) cancer cells. HNTs-DOX-FA significantly lowers the IC_50_ value (~5 μg/mL after 24 h) compared to non-targeted DOX, via a mechanism based on folate receptor binding and pH-dependent release [37].

In the context of multidrug resistance (MDR), such targeted, stimulus-responsive delivery is particularly important, as it increases the accumulation of drugs within tumour cells that overexpress efflux pumps (e.g., P-gp, MRP1), while minimising systemic exposure and off-target effects. By releasing the drug within acidic tumour microenvironments or intracellular compartments such as endosomes and lysosomes, HNT-based systems can bypass drug efflux mechanisms and maintain cytotoxic concentrations at the target site.

Systems that employ transferrin and PEG can increase tumour retention and penetration, as well as prolong circulation time, and enhance cytotoxic effects in vivo in mice relative to free DOX [38]. The use of aptamers enables specific recognition of CCRF-CEM T-cell leukaemia cells through interaction with protein tyrosine kinase 7 (PTK7) receptors [39].

HNTs can also be combined with bioactive carriers such as chitosan to improve stability and achieve pH-controlled release. In this dual-nanocarrier system, HNTs provide structural support and loading capacity, while chitosan improves responsiveness to acidic environments. The HNT–chitosan–khellin complex showed enhanced drug release at pH 5.5, indicating its suitability for applications requiring selective activation in acidic environments [40]. Although the HNT–chitosan–khellin system demonstrated promising pH-responsive release properties, further biological studies, including cellular assays, are necessary to validate its efficacy and safety in targeted anticancer applications.

Various HNT functionalisation strategies, such as surface chemical modifications, conjugation with targeting ligands, cytotoxic loading, and carrier support (e.g., chitosan, PEG, CuO), enable the versatile use of these nanocarriers in cancer therapy. Compared to other drug delivery systems, HNTs offer the advantages of smaller size, high chemical stability, and broad functionalisation potential, making them highly attractive for future clinical applications.

### 4.3. Biocompatibility and Toxicity of Halloysite Nanotubes

The biological safety of HNTs is critical for their use in biomedical applications, particularly in targeted therapies and drug delivery systems. Available data suggest that HNTs are highly biocompatible across a wide concentration range and biological models. However, toxicity may vary depending on dose, exposure time, and chemical composition.

Studies using mouse macrophages (Raw 264.7) showed no cytotoxicity at concentrations up to 250 µg/mL [41]. MTT viability assays revealed low toxicity to human cervical carcinoma (HeLa) and human liver (HepG2) cell lines at concentrations up to 100 µg/mL. However, significant viability loss was observed at 600 µg/mL after 72 h. This effect was particularly pronounced for HNT-U and HNT-S samples, which were obtained, respectively, from a U.S. deposit and material with high iron and titanium oxide content. Samples were characterised by higher surface charge and shorter tube length, which may account for their elevated toxicity [42]. Halloysite toxicity was tested in eukaryotic, multi-organ, transparent nematode Caenorhabditis elegans, where it localised exclusively in the digestive tract and did not induce significant toxicity [43]. In vivo acute and chronic toxicity tests in Bagg Albino Laboratory-Bred / c subline (BALB/c) mice, using oral and intraperitoneal administration for 14 and 90 days, showed no significant behavioural, morphological, or histopathological changes at doses of up to 5 mg/kg [41]. Dermal tests using HNT-based transdermal systems with metoclopramide showed no inflammatory response or irritation, confirming their potential as drug carriers across the skin barrier [44]. Furthermore, studies using zebrafish embryos revealed that halloysite did not affect morphological development at concentrations of up to 25 mg/mL. It accumulated only in the gastrointestinal tract, with its concentration decreasing over time [45]. Furthermore, the CBMN (cytokinesis-block micronucleus) assay on human liver cell line (HepG2) revealed an increase in micronuclei formation only at the highest concentration (100 µg/mL) after 24h, indicating moderate genotoxicity with prolonged exposure [42].

## 5. Phytocannabinoids

**Figure 2:**
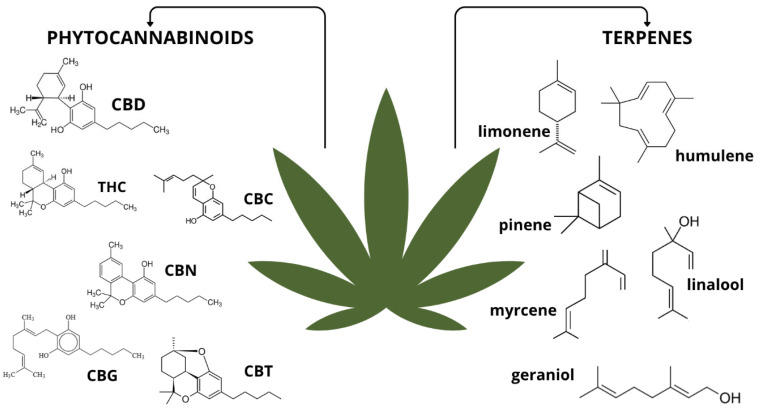
Classification of substances isolated from *Cannabis sativa* L. with examples. Abbreviations: CBD—cannabidiol; THC—tetrahydrocannabinol; CBC—cannabichromene; CBN—cannabinol; CBG—cannabigerol; CBT—cannabicitran. Figure created by the authors.

In recent years, cannabis has attracted increasing attention as a supportive therapy for cancer patients. The dried flowers and oils of *Cannabis sativa* L. contain a complex mixture of bioactive phytochemicals, particularly phytocannabinoids and terpenes. The qualitative and quantitative composition of these compounds varies depending on the plant strain, cultivation conditions, and geographic origin, all of which can significantly affect their pharmacological properties [46,47]. Figure 2 shows a schematic classification of the main phytochemicals isolated from cannabis.

Over 120 phytocannabinoids have been identified, including non-psychoactive ones such as cannabidiol (CBD), cannabigerol (CBG), and cannabichromene (CBC). These phytocannabinoids exhibit anti-inflammatory, antiproliferative, and neuroprotective properties but do not have the psychoactive effects of tetrahydrocannabinol (THC) [48,49]. In addition to phytocannabinoids that occur naturally in *Cannabis sativa* L., there are also synthetic cannabinoids, which are chemically synthesised analogues. Furthermore, endogenous cannabinoids (endocannabinoids) are produced within the human body. The most thoroughly characterised endocannabinoids are anandamide (AEA) and 2-arachidonoylglycerol (2-AG). These molecules function as part of the endocannabinoid system (ECS), which is an evolutionarily conserved signalling network involved in regulating key physiological processes such as pain perception, immune response, energy balance, mood, and homeostasis [50,51,52]. The ECS comprises cannabinoid receptors (primarily CB1 and CB2), endogenous ligands (AEA, 2-AG), and enzymes responsible for their synthesis and degradation. CB1 receptors are known to be expressed in high quantities within the central nervous system, particularly in the brain. However, they are also present in peripheral tissues, including the lungs, heart, liver, gastrointestinal tract, skeletal muscle, and adipose tissue. CB2 receptors are predominantly located on immune cells, as well as in the spleen, thymus, and lymph nodes. More limited expression of CB2 receptors has been observed in the brain and other peripheral organs [53,54].

Interacting with the endocannabinoid system’s CB1 and CB2 receptors modulates other signalling pathways that regulate survival, proliferation, and apoptosis. Preclinical studies, including cellular and animal models, suggest that phytocannabinoids and their derivatives can inhibit tumour progression by modulating the tumour microenvironment, regulating the cell cycle, and inducing cell death [48,55,56,57].

Despite our growing understanding of the pharmacokinetics and mechanisms of action of CBD, further research on lesser-known cannabinoids such as CBG and CBC is crucial to fully evaluate their therapeutic potential and safety [58].

### 5.1. Therapeutic Applications of Phytocannabinoids in Lung Cancer

**Figure 3:**
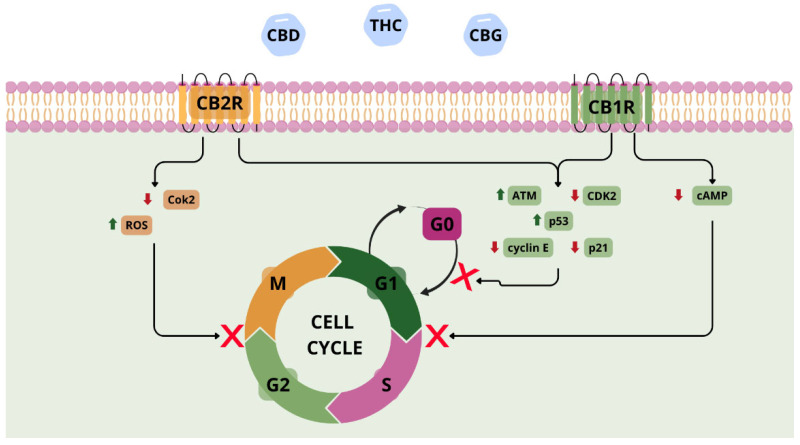
Involvement of CB1R and CB2R receptors and phytocannabinoids in the mechanism of cell cycle inhibition. Abbreviations: CBD—cannabidiol; THC—tetrahydrocannabinol; CBG—cannabigerol; ATM—ataxia telangiectasia; ROS—reactive oxygen species; cAMP—cyclic adenosine monophosphate. Figure created by the authors.

Phytocannabinoids exhibit promising antitumour properties and are being investigated intensively as a potential treatment for lung cancer. Preclinical studies (using cell lines and animal models) have demonstrated their effects on proliferation, autophagy, apoptosis, angiogenesis, and cancer cell migration [59]. Phytocannabinoids act mainly via CB1 and CB2 receptors and modulate oncogenic signalling pathways such as phosphoinositide 3-kinase/protein kinase B (PI3K/AKT) and mitogen-activated protein kinase (MAPK) cascades [60,61]. However, they can also activate transient receptor potential vanilloid 1 (TRPV1) and inhibit G protein-coupled receptor 55 (GPR55), inducing endoplasmic reticulum stress, leading to cancer cell death independently of canonical cannabinoid receptors [59,60]. In NSCLC models (A549 and SW-1573 cell lines), cannabinoids inhibited growth and migration. Preet, A. et al. demonstrated that agonists of CB1/CB2 (e.g., WIN55,212-2, JWH-015) exhibited antitumour effects that were partially reversed by antagonists (AM251, AM630), which confirms the role of the endocannabinoid system in tumour regulation [61]. Selective CB2R agonists, which have a better safety profile, inhibited in vivo without affecting healthy cells [60]. Combining CBD with cytostatics (e.g., etoposide) increases the therapeutic efficacy of the cytostatic drug via inhibition of the PI3K/AKT/mTOR pathway and activation of p53-dependent autophagy in NSCLC cells. Inhibition of this signalling cascade suppresses proliferative and survival signals, sensitizing tumour cells to cytotoxic stress, while p53 activation promotes autophagic mechanisms that, under these conditions, contribute to programmed cell death. This effect appears specific to CBD, as other phytocannabinoids such as THC did not enhance etoposide efficacy, potentially due to divergent interactions with intracellular signalling networks [62]. Combining CBD with the chemotherapy drug doxorubicin has also been shown to reduce the formation of lung metastases in a mouse model of breast cancer [63]. Phytocannabinoids may also modulate the tumour microenvironment by inhibiting neutrophil extracellular trap (NET) formation, a mechanism involved in metastasis [64]. Moreover, phytocannabinoids can interfere directly with the cell cycle of cancer cells, leading to arrest at various checkpoints (Figure 3) [65,66,67,68].

Importantly, beyond their receptor-mediated effects, phytocannabinoids may contribute to overcoming multidrug resistance (MDR) by targeting tumour metabolic reprogramming, a hallmark of cancer that supports survival and progression. Cancer cells often rely on aerobic glycolysis, de novo lipid biosynthesis, and glutamine-dependent anaplerosis—processes that are tightly regulated by signalling pathways such as ROS, AMP-activated protein kinase (AMPK), mitogen-activated protein kinases (MAPK), phosphoinositide 3-kinase (PI3K), hypoxia-inducible factor 1-alpha (HIF-1α), and the tumour suppressor protein p53. Phytocannabinoids have been shown to modulate these pathways, thereby disrupting the metabolic adaptability of tumour cells and sensitising them to therapeutic stress. This can limit the availability of critical nutrients and energy for resistant cancer cells, potentially restoring their susceptibility to treatment. This suggests that phytocannabinoids alone, without concurrent cytotoxic chemotherapy, might suppress tumour growth by regulating energy metabolism and oncogenic signalling in combination, offering a complementary or alternative strategy to conventional drugs [15].

Clinical reports suggest that the use of cannabinoid-based products improve the quality of life of lung cancer patients by reducing pain, improving appetite, and alleviating nausea [62,69]. The use of cannabis and/or cannabinoids alongside antiemetic therapy (in treatment regimens consistent with established guidelines) has been shown to increase the effectiveness of chemotherapy-induced antiemetic therapy, especially in cases of refractory nausea and vomiting [70]. However, the American Society of Clinical Oncology (ASCO) guidelines advise physicians against using cannabis or cannabinoids to treat cancer, except in clinical trials [71]. Despite the proven therapeutic potential of medical marijuana in oncology, critical attention should be paid to the method of administration and product quality. Some studies have indicated a potential correlation between smoking cannabis and an increased risk of certain lung cancers, particularly adenocarcinoma and large-cell neuroendocrine carcinoma [72,73]. These findings emphasise the need for caution when implementing cannabis-based therapies, especially outside of standardised medical protocols. Furthermore, illegal cannabis products are often mixed with tobacco to reduce costs, and their chemical composition is neither regulated nor well-characterised, posing additional health risks. The route of administration also plays a significant role in safety and therapeutic efficacy. Medical cannabis should be administered using certified vaporisation devices. The use of such devices allows for the regulation of dosage and, at the same time, prevents thermal degradation of the plant material [74,75]. This minimises the inhalation of harmful combustion by-products.

In order to capitalise on the therapeutic potential of cannabinoids in oncology, clinical applications must rely on pharmacologically characterised preparations and validated delivery devices. Ensuring such controlled conditions will allow cannabis-based therapies to be recognised as safe and effective for lung cancer patients.

### 5.2. Advances in Nanocarrier Systems for Cannabinoid Delivery

Phytocannabinoids exhibit multifaceted biological activity, including cytotoxic, antiproliferative, pro-apoptotic, and immunomodulatory effects. This makes them promising candidates for anticancer therapy. However, their clinical application is currently limited by their poor pharmacokinetics, including low water solubility, variable bioavailability, short half-life, first-pass hepatic metabolism, and psychoactive effects in the case of THC [27].

Encapsulation in nanocarriers can increase solubility and maintain high local concentrations at the site of administration. When administered to the lungs (the preferred method for treating lung cancer), the carriers enable the drug to be deposited deep within the lungs, allowing it to be rapidly absorbed by the epithelium and bypassing first-pass metabolism in the liver.

Preclinical studies have shown that such strategies can significantly improve pharmacokinetic parameters: for example, elastic proteinoid nanoparticles increased the bioavailability of orally administered CBD by 135–170% and extended the plasma half-life from minutes to hours in rats [76]. Another study found that self-emulsifying drug delivery systems (SNEDDSs) increased the maximum plasma concentration (Cmax) and total drug exposure (AUC) of CBD compared to oil formulations [77].

Nanoformulations such as liposomes, lipid nanoparticles, polymeric nanoparticles, emulsions, and hybrid systems improve cannabinoid solubility. They can also enable controlled, targeted delivery to tumour tissues [26,27]. For instance, nanoemulsions containing CBD have demonstrated stronger cytotoxic effects against breast cancer cells than free cannabidiol, whilst reducing toxicity toward healthy cells [78]. Similarly, cannabinoid-loaded nanoliposomes have shown enhanced blood–brain barrier penetration and greater tumour accumulation in glioblastoma models [78]. Combining cannabinoids with nanocarriers has been shown to selectively target cancer cells, as demonstrated by in vitro studies conducted on glioblastoma cells. Combining CBG, a nanocarrier, and a tubulin-targeting drug disrupted the microtubules of cancer stem cells, preventing further cell division [79]. Nanocarriers enable the combined delivery of cannabinoids and conventional chemotherapeutics. In studies by Taudul et al., iron oxide nanoparticles were developed as potential carriers for cannabidiol (CBD). When co-administered with doxorubicin in lung cancer cells (A549), they were found to enhance anticancer efficacy compared to either agent alone [80]. Simultaneously delivering cannabinoids and chemotherapeutic agents may increase therapeutic efficacy at lower drug doses, thereby reducing the risk of adverse effects. Innovative delivery systems that respond to the tumour microenvironment (e.g., pH, enzymes, temperature) can further enhance therapeutic selectivity by allowing “smart” release of cannabinoids at disease-specific sites [80,81]. Using targeting ligands, such as monoclonal antibodies or molecules that recognise CB1 and CB2 receptors, can facilitate precise delivery to cancer cells [82,83]. Furthermore, nanostructured cannabinoid formulations may provide palliative treatment for cancer patients to alleviate symptoms such as pain, nausea, cachexia, and anorexia. Using nanoformulations enables greater precision in dosing and optimisation of therapeutic outcomes, which could potentially improve patients’ quality of life [71,84].

Despite the availability of encouraging preclinical data, there is still limited clinical evidence regarding nanocarrier-based cannabinoid therapy. To date, most studies have been conducted in vitro or on animal models. Therefore, although the improvements in pharmacokinetics observed in preclinical and early clinical studies are promising, further translational research and large-scale clinical trials are needed to confirm whether these enhancements can consistently be achieved in the treatment of cancer patients [85]. Standardised methods for synthesis, validation, and quality control of cannabinoid-loaded nanocarriers will also be crucial to their successful clinical implementation.

## 6. Discussion and Perspectives: Positioning HNT-Based Phytocannabinoid Systems in Lung Cancer Therapy

Nanotechnology has emerged as a transformative approach in oncology, enabling the development of targeted drug delivery systems that improve the therapeutic index and minimise systemic toxicity. In the treatment of lung cancer, where conventional chemotherapies such as taxanes and platinum derivatives are often limited by resistance mechanisms and side effects, the integration of advanced nanocarrier platforms offers a promising way forward for more effective and less harmful therapies. This review focuses on the intersection of two complementary fields: natural clay nanotubes (specifically halloysite nanotubes—HNTs) and phytocannabinoids. This novel yet under-explored strategy could be used to manage lung cancer.

### 6.1. The Comparative Advantages of Halloysite Nanotubes (HNTs) over Established Nanocarriers

HNTs possess a distinctive combination of structural and chemical characteristics that make them uniquely suited to delivering hydrophobic and chemically sensitive agents, such as phytocannabinoids. Their naturally occurring tubular morphology provides a high aspect ratio and dual-surface chemistry, with a positively charged, alumina-rich inner lumen and a negatively charged, silica-rich outer surface [30]. This duality enables the orthogonal loading of multiple payloads; for instance, a phytocannabinoid and a chemotherapeutic agent can be loaded separately. This dual-compartment functionality cannot be easily achieved with conventional nanocarriers such as liposomes or spherical polymeric nanoparticles.

In addition, HNTs exhibit superior physicochemical stability. Unlike liposomes, which are susceptible to lipid oxidation, aggregation and premature drug leakage during storage—even when refrigerated—HNTs maintain their structural integrity over extended periods and are resistant to both chemical and enzymatic degradation in physiological environments. The synthesis of polymeric nanoparticles often involves complex multi-step processes using organic solvents, which leads to high production costs, scalability challenges and the potential generation of toxic by-products. In contrast, HNTs are inexpensive, naturally abundant, and can be processed without hazardous solvents, facilitating cost-effective, large-scale production.

HNTs can tolerate standard sterilisation procedures, such as autoclaving and gamma irradiation, without compromising the stability of their cargo. This is a significant advantage for clinical manufacturing. Their outer surface can be easily modified with targeting ligands, such as antibodies, peptides, or small molecules, to improve selective accumulation in malignant tissues. These attributes collectively establish HNTs as a robust, versatile, and economically viable nanocarrier platform, offering clear advantages over other nanotechnologies for applications requiring targeted, stable and scalable drug delivery solutions.

### 6.2. Limitations of Other Nanotechnologies and How HNTs Address These Limitations

Despite their widespread use, other nanocarrier systems have limitations that can restrict their clinical application. For example, although liposomes are effective at enhancing the bioavailability of hydrophobic agents, they have a short shelf life and are sensitive to temperature and pH fluctuations [86]. Meanwhile, polymeric nanoparticles often face scalability issues and batch-to-batch variability. Both can release their payload prematurely in the bloodstream, reducing drug accumulation at the tumour site. HNTs mitigate these limitations through their rigid aluminosilicate architecture, which enables precise control over release kinetics via surface chemistry modification. Their stability under physiological pH levels and minimal degradation during circulation increases the likelihood that therapeutic payloads will reach their intended targets at effective concentrations.

### 6.3. Safety and Immunogenicity

Several in vitro and in vivo studies have supported the biocompatibility of HNTs, reporting minimal activation of the complement cascade, low cytokine induction, and negligible cytotoxicity towards non-malignant cells at therapeutically relevant doses. Biodistribution studies reveal primary accumulation in the liver and spleen following intravenous administration, with gradual clearance over time [41,42]. However, the long-term fate of aluminosilicate structures in vivo, particularly following repeated dosing, requires further evaluation. While these findings are encouraging, there is a lack of systematic studies investigating the specific effects of HNTs on immune system modulation, including potential long-term immunotoxicity. This remains a key issue to be addressed before clinical translation can be considered.

### 6.4. Clinical Translation Potential

The pathway from concept to clinic for HNT/phytocannabinoid systems requires coordinated formulation optimisation, preclinical validation, and regulatory-compliant manufacturing. The first step is to achieve high encapsulation efficiency and predictable release under physiological and tumour-mimicking conditions. This should be followed by the selection of targeting ligands that are matched to biomarkers that are prevalent in specific lung cancer subtypes, such as the overexpressed CB2 receptor or EGFR variants. Lung cancer models should then be used to evaluate the pharmacokinetics, biodistribution, tumour uptake, and therapeutic efficacy of the system, both as a monotherapy and in combination with reduced doses of standard chemotherapeutics. Comprehensive safety profiling, including repeated-dose toxicity, immunotoxicity, genotoxicity, and clearance, must be performed in parallel. Manufacturing processes must meet Good Manufacturing Practice (GMP) standards to ensure consistency of batches, control of ligand density, and an endotoxin-free status. Although no HNT-based phytocannabinoid formulation has yet entered clinical trials, there is sufficient preclinical evidence for the individual components to justify targeted investment in translational development.

### 6.5. Concluding Perspective

Combining the structural advantages of HNTs with the multifaceted therapeutic potential of phytocannabinoids provides a logical and creative approach to treating lung cancer. Overcoming the limitations of conventional nanocarriers and addressing the pharmacokinetic and delivery challenges of phytocannabinoids could enable HNT-based systems to improve therapeutic outcomes, reduce systemic toxicity and enhance patients’ quality of life. Future research should focus on optimising formulation parameters, validating efficacy in relevant preclinical models and establishing a robust safety profile, in order to support clinical translation. This convergence of two promising fields not only represents an unexplored research opportunity, but also a potential step forward in developing more effective, targeted, and patient-centred lung cancer therapy strategies.

To date, nanotechnology research has revealed the significant potential of targeted drug delivery systems to minimise systemic toxicity in oncology, including in the treatment of lung cancer. In this context, two distinct areas of research—natural nanomaterials, such as halloysite nanotubes (HNTs), and phytocannabinoids, which have been documented to have anticancer activity—represent a logical yet currently unexplored integration point for a novel class of therapeutic systems.

HNTs, with their natural tubular morphology, biocompatibility, and modifiability, have demonstrated considerable versatility as carriers of anticancer and anti-inflammatory agents. Utilising these materials to encapsulate salicylic acid, curcumin, and cisplatin has laid the groundwork for developing sustained-release systems characterised by high chemical stability.

**Figure 4:**
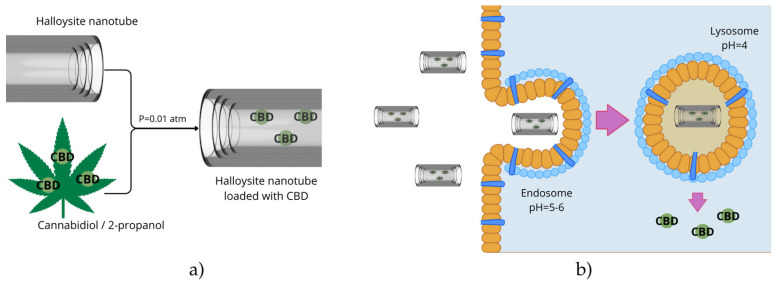
Proposal for incorporating halloysite nanotubes (HNT) with phytocannabinoids, using cannabidiol (CBD) as an example (**a**), and putative release in lung-cancer cells (**b**). Figure created by the authors.

To date, no studies have been found on the direct encapsulation of phytocannabinoids within halloysite structures. However, findings from the encapsulation of essential oils, phenolic compounds, and flavonoids suggest that halloysite nanotubes (HNTs) are well-suited to such a process, from both physicochemical and pharmacological perspectives. Figure 4 presents a conceptual schematic illustrating a proposed strategy for CBD loading into HNTs and its release within lung cancer cells.

Combining the anticancer potential of cannabinoids with the controlled delivery capacity of HNTs opens up new possibilities for intelligent therapeutic systems in the treatment of lung cancer. HNTs could act both as passive carriers, protecting cannabinoids from degradation, and as active targeting tools through appropriate surface functionalisation. Systems responsive to tumour microenvironment cues (e.g., pH, metalloproteinase activity) could minimise side effects further and enhance treatment efficacy. Therefore, there is a clear need for in-depth research, including the development of effective methods for incorporating cannabinoids into HNTs, assessing their stability in biological systems, and assessing their antitumour efficacy in in vitro and in vivo lung cancer models. Toxicological, pharmacokinetic, and immunological analyses will be necessary to assess the translational potential of these systems for clinical applications.

In conclusion, combining cannabinoids with halloysite nanotubes is a logical and rational approach that builds on current trends in personalised oncology, but one that is underutilised. This approach could improve the efficacy of anti-cancer treatments and contribute to the development of safer, more sustainable lung cancer treatment methods.

## Figures and Tables

**Table 1:** Comparison of targeted nanocarrier platforms investigated for lung cancer treatment.

Nanocarrier Type	Size Range (nm)	Key Components	Targeting Strategy	Advantages	Limitations	Representative Application	FDA Approval Status	Patient Outcome
Polymeric Nanoparticles	50–200	PLA, PLGA, Chitosan	Active (EGFR, integrins), Passive (EPR)	Controlled release, biocompatibility	Limited stability in circulation	PLGA-siRNA for NSCLC [26]	Not FDA approved	N/A
Liposomes	80–150	Phospholipids, cholesterol	EGFR, transferrin, Passive (EPR)	Low toxicity, enhanced bioavailability	Risk of leakage or degradation	Liposomal DOX, Oxaliplatin [27]	Liposomal DOX FDA approved for other cancers; NSCLC indication under clinical trials	Clinical: improved drug tolerability; variable efficacy data
Solid Lipid Nanoparticles (SLNs)	50–180	Lipids with high melting points	Similar to liposomes	Higher stability, better PK profile	Limited drug loading capacity	SLN-Paclitaxel [26]	Not FDA approved for NSCLC; some formulations approved for other indications	Preclinical: enhanced paclitaxel delivery, reduced systemic toxicity
Metallic Nanoparticles	10–100	AuNPs, AgNPs	Surface ligand-conjugated	Photothermal properties, imaging compatibility	Potential toxicity, accumulation risk	Photothermal therapy [28]	Not FDA approved; in clinical research	Early clinical trials: improved local tumour control; limited survival data
Hybrid Organic–Inorganic	50–150	Polymer + metal core/shells	Dual (EGFR + integrins)	Multimodal targeting, synergistic therapy	Complex synthesis	Chemo-photothermal hybrid systems [29]	Not FDA approved	N/A
Biomimetic Nanocarriers	100–200	Cancer/immune cell membrane-coated particles	Homotypic targeting, immune evasion	Personalised therapy, immune modulation	Standardisation and immunogenicity	Cancer cell-membrane-coated NPs [22]	Not FDA approved	N/A
TME-Responsive Nanocarriers	50–120	pH-, ROS-, or hypoxia-sensitive polymers	Stimuli-triggered	High specificity, controlled drug release in the tumour niche	Dependent on precise TME conditions	ROS-responsive micelles, pH-sensitive NPs [30]	Not FDA approved	N/A

N/A—not applicable.

## Data Availability

No new data were created or analysed in this study. Data sharing is not applicable to this article.
